# Community dissemination of blaCTX-M-dominant extended-spectrum β-lactamase-producing Escherichia coli with widespread multidrug resistance in domestic cats from Ecuador: A One Health molecular epidemiological study

**DOI:** 10.14202/vetworld.2026.3595-3607

**Published:** 2026-08-17

**Authors:** Morales Gerardo, Sánchez Raquel, Gualán Karen, Vitonera Rogel, González Janzel, Luna Johon, Ponce Natasha, Alejandro Ronny, Sánchez Robert

**Affiliations:** 1 Master's Program in Veterinary Medicine with a Specialization in Small Animal Clinical Medicine and Surgery, Universidad Técnica de Machala, Machala, El Oro, Ecuador.; 2 School of Biochemistry and Pharmacy, Universidad Técnica de Machala, Machala, El Oro, Ecuador.; 3 School of Veterinary Medicine, Universidad Técnica de Machala, Machala, El Oro, Ecuador.; 4 Universidad Autónoma de Madrid and School of Veterinary Medicine, Universidad Técnica de Machala, Machala, El Oro, Ecuador.; 5 Graduate Program in Animal Health in the Amazon, Universidade Federal do Pará, Castanhal, Pará, Brazil.; 6 Instituto Nacional de Investigación en Salud Pública "Dr. Leopoldo Izquieta Pérez" (INSPI), Reference Center for Antimicrobial Resistance CZ6, Cuenca, Azuay, Ecuador.

**Keywords:** antimicrobial resistance, companion animals, Ecuador, Escherichia coli, extended-spectrum β-lactamase, multidrug resistance, One Health, blaCTX-M

## Abstract

**Background and Aim:**

Extended-spectrum β-lactamase (ESBL)-producing *Escherichia coli* represents an increasing One Health threat because of its dissemination among humans, animals, and the environment and its frequent association with multidrug resistance (MDR). Companion animals may serve as reservoirs of clinically important resistant bacteria; however, molecular epidemiological information on ESBL-producing *E. coli* in domestic cats from Ecuador remains limited. This study aimed to determine the prevalence, antimicrobial susceptibility patterns, β-lactamase gene distribution, and potential risk factors associated with ESBL-producing *E. coli* carriage in domestic cats from Machala, Ecuador.

**Materials and Methods:**

A cross-sectional study was conducted from August 2023 to August 2024 involving 199 domestic cats presented to the Veterinary Clinic of the Universidad Técnica de Machala. Rectal swabs were cultured on CHROMagar™ ESBL, and ESBL production was phenotypically confirmed using the double-disk synergy test according to Clinical and Laboratory Standards Institute criteria. Antimicrobial susceptibility testing against 12 agents was performed using the Kirby–Bauer disk diffusion method. Polymerase chain reaction was used to detect , , and . MDR was defined as non-susceptibility to at least one antimicrobial agent in three or more categories. Risk factor associations were evaluated using Pearson’s chi-square or Fisher’s exact test.

**Results:**

ESBL-producing *E. coli* was detected in 83 of 199 cats, corresponding to a prevalence of 41.7% (95% confidence interval: 35.1–48.7). The gene predominated in 60/83 isolates (72.3%), followed by in 19/83 (22.9%) and in 1/83 (1.2%). At least one targeted gene was detected in 64/83 isolates (77.1%), whereas 19/83 phenotypically positive isolates lacked all three targeted genes. MDR was observed in 74/83 isolates (89.2%). All isolates remained susceptible to imipenem and meropenem, whereas the highest resistance rates were recorded for trimethoprim–sulfamethoxazole, tetracycline, cefepime, levofloxacin, and doxycycline. None of the evaluated risk factors was significantly associated with ESBL gene positivity.

**Conclusion:**

Domestic cats in Machala showed a high prevalence of -dominant, multidrug-resistant ESBL-producing *E. coli*. These findings indicate widespread community dissemination and support the inclusion of companion animals in Ecuadorian antimicrobial resistance surveillance and stewardship programs under a coordinated One Health framework.

## INTRODUCTION

Antimicrobial resistance (AMR) is recognized by the World Health Organization as one of the ten leading global public health threats, with an estimated 4.95 million deaths associated with bacterial AMR worldwide in 2019 [1]. Among members of the order *Enterobacterales*, the dissemination of extended-spectrum β-lactamase (ESBL)-producing *Escherichia coli* represents a major public health concern because these enzymes confer resistance to third- and fourth-generation cephalosporins and are frequently co-associated with resistance determinants against fluoroquinolones, tetracyclines, aminoglycosides, and folate pathway inhibitors, thereby substantially limiting therapeutic options and often leaving carbapenems as the last-line treatment for severe infections [2, 3]. Among ESBL determinants, *bla*_CTX__-M_, particularly the CTX-M-15 and CTX-M-14 variants, has driven the global emergence of community- and hospital-acquired ESBL infections during the past two decades. Although *bla*_TEM_ and *bla*_SHV_ are less frequently associated with cefotaximase-producing phenotypes than *bla*_CTX__-M_, they remain important contributors to the ESBL gene pool through clinically relevant variants such as *bla*_TEM-52_ and *bla*_SHV-12_ [4, 5]. Within the One Health framework, these resistance genes and their associated clonal lineages circulate among humans, food-producing animals, companion animals, and environmental reservoirs, highlighting the interconnected nature of AMR transmission. Increasing evidence indicates that domestic pets living in close physical contact with their owners and sharing the household microbiome can serve as reservoirs and potential disseminators of clinically important ESBL-producing *E. coli* [6, 7].

Companion cats represent an understudied but epidemiologically important reservoir for ESBL-producing *E. coli*. Compared with dogs, cats exhibit grooming behaviors that facilitate fecal–oral transmission of resistant bacteria within households, while their frequent indoor–outdoor movement increases opportunities for environmental acquisition and dissemination [8, 9]. Reported intestinal carriage of ESBL-producing *E. coli* in domestic cats varies considerably across geographic regions, with pooled global prevalence estimates of approximately 5%, increasing to 16.8% and 16.6% in Asian and African feline populations, respectively [7]. Even higher prevalences have been reported in clinic-based surveys from Latin America, including Brazilian studies documenting ESBL carriage rates of approximately 44% and multidrug resistance (MDR) exceeding 90% [10]. Across these investigations, *bla*_CTX-M_ has consistently been identified as the predominant ESBL determinant in feline isolates [7, 11]. In Ecuador, however, molecular epidemiological evidence remains limited. Gonzales-Zubiate *et al.* [12] characterized pandemic *bla*_CTX-M_ variants (CTX-M-14, CTX-M-27, CTX-M-55, and CTX-M-65) in companion animals from Quito within a One Health framework. Nevertheless, comparable information from other regions of Ecuador remains unavailable.

Despite the increasing recognition of companion animals as reservoirs of antimicrobial-resistant bacteria, substantial knowledge gaps remain. To date, no molecular epidemiological study has specifically investigated intestinal ESBL-producing *E. coli* in domestic cats from Ecuador, particularly in the coastal province of El Oro. Furthermore, no study has comprehensively evaluated the prevalence of ESBL-producing *E. coli*; the distribution of the major ESBL genes, *bla*_CTX-M_, *bla*_SHV_, and *bla*_TEM_; antimicrobial susceptibility patterns; MDR profiles; and epidemiological risk factors within the same feline population. This lack of evidence limits understanding of the contribution of domestic cats to the ecology and transmission dynamics of AMR at the human–animal–environment interface and hinders the development of evidence-based One Health surveillance and antimicrobial stewardship strategies in Ecuador. Given intensive livestock production, aquaculture, increasing urbanization, and frequent interactions among humans, companion animals, food-producing animals, and the environment in El Oro Province, investigating ESBL-producing *E. coli* in domestic cats is particularly important for understanding the regional dissemination of antimicrobial-resistant bacteria.

Therefore, this study aimed to determine the prevalence of intestinal ESBL-producing *E. coli* among domestic cats attending a referral Veterinary Clinic in Machala, Ecuador, and to characterize their antimicrobial susceptibility profiles, MDR patterns, and the distribution of the *bla*_CTX-M_, *bla*_SHV_, and *bla*_TEM_ genes. In addition, owner-reported demographic and management factors potentially associated with ESBL carriage were evaluated to improve understanding of the epidemiology of antimicrobial-resistant *E. coli* in companion cats. We hypothesized that domestic cats from Machala would harbor intestinal ESBL-producing *E. coli* at a prevalence comparable to or greater than that reported in other Latin American companion animal populations, with *bla*_CTX-M_ as the predominant ESBL determinant. We further hypothesized that conventional owner-reported risk factors, including previous antimicrobial use, hospitalization, and raw feeding, would not fully explain ESBL carriage, supporting widespread community-level dissemination rather than transmission driven solely by clinical exposures.

## MATERIALS AND METHODS

### Ethical approval

The study protocol was reviewed and approved by the Comité de Ética para Experimentación con Animales (CEEA) of the Universidad Técnica de Machala under approval number UTMACH-CEEA-2025-035-AC. The protocol was also registered as an institutional research project under resolution Res. 244-2024-CU-SO-13. The ethical review process adhered to national and international standards, including the principles of the 3Rs (Replacement, Reduction, and Refinement), ensuring that animal use was justified, minimized, and conducted with the highest standards of welfare. Rectal swab sampling was performed by trained veterinarians using gentle, non-invasive techniques to minimize stress and discomfort to the cats. No invasive procedures, sedation, or additional interventions beyond routine clinical handling were involved. Written informed consent was obtained from each cat owner prior to participation, after a detailed explanation of the study objectives, procedures, potential risks (none anticipated beyond minimal handling stress), and the right to withdraw at any time without affecting the animal’s clinical care. All data were handled confidentially, and participating animals received standard veterinary care as required. The study followed the STROBE guidelines for reporting observational studies [13]. The study complied fully with Ecuadorian regulations on animal experimentation and the ARRIVE 2.0 guidelines for reporting animal research [14].

### Study period and location

The study was conducted between August 2023 and August 2024 at the Veterinary Clinic of the Universidad Técnica de Machala (UTMACH), Machala, El Oro, Ecuador.

### Study design

A cross-sectional observational study was conducted to characterize intestinal carriage of ESBL-producing *E. coli* in domestic cats.

### Animals, sampling frame and sample size

A non-probabilistic consecutive convenience sampling strategy was used, based on the inclusion of domestic cats (*Felis catus*) that attended the Veterinary Clinic of the Universidad Técnica de Machala between August 2023 and August 2024. This strategy was selected because of the observational and cross-sectional nature of the study, as well as the availability of animals attended during the research period. In total, 199 cats were included.

Domestic cats of any age, sex, and breed were eligible, provided that they met the following inclusion criteria: (1) attendance at the clinic during the study period, either for a general check-up, vaccination, routine wellness examination, or clinical treatment; (2) written informed consent signed by the owner; and (3) feasibility of rectal sampling without evident risk to the animal. Cats whose owners did not authorize participation were excluded.

The cohort was divided into two groups according to the reason for clinical attendance. The first group included animals that presented for vaccination or routine check-up and were operationally classified as “apparently healthy,” provided that they had not received antimicrobial therapy during the seven days preceding sampling. The second group included animals that presented for clinical treatment; these cats were sampled regardless of recent antimicrobial exposure to enable sentinel surveillance of cats potentially exposed to clinical factors associated with the carriage of resistant bacteria.

The final sample size was 199 cats. No formal statistical power analysis was performed because the study had an exploratory, descriptive design, primarily aimed at estimating the frequency of intestinal carriage of ESBL-producing *E. coli* among cats attending a referral veterinary clinic. Nevertheless, the number of animals included represents all cats that met the selection criteria during the sampling period and whose owners authorized participation.

### Bacterial isolation and phenotypic ESBL confirmation

Rectal swabs were streaked using the composite streaking method onto selective chromogenic agar plates (CHROMagar™ ESBL, CHROMagar, Paris, France), designed to isolate ESBL-producing bacteria while concomitantly inhibiting the accompanying microbiota. The plates were incubated at 35 ± 2 °C for 18–24 h. Red-colored colonies were considered presumptive ESBL-producing *E. coli*.

Suspected colonies were subcultured on Mueller–Hinton agar and subjected to Gram staining, as well as oxidase and catalase tests. Isolates corresponding to Gram-negative, oxidase-negative bacilli were selected for definitive identification using the Enterosystem 18R system (this system allows the biochemical characterization of different genera within the family *Enterobacteriaceae*, including *Escherichia*, *Enterobacter*, *Klebsiella*, *Citrobacter*, *Salmonella*, *Proteus*, *Serratia*, *Yersinia*, and *Arizona*). Bacterial suspensions were prepared in sterile saline solution, inoculated into the system wells, and incubated at 36 ± 1 °C according to the manufacturer’s instructions.

Phenotypic confirmation of ESBL production was performed by adjusting the bacterial concentration to 1.5 × 10⁸ colony-forming units (CFU)/mL, equivalent to the 0.5 McFarland standard. The disk diffusion method was applied on Mueller–Hinton agar, following the recommendations of the Clinical and Laboratory Standards Institute (CLSI, document M100). Disks of ceftazidime 30 µg, cefotaxime 30 µg, ceftazidime/clavulanic acid 30/10 µg, and cefotaxime/clavulanic acid 30/10 µg were used.

ESBL production was interpreted when an increase of ≥5 mm in the inhibition zone was observed between the antibiotics alone and their combinations with clavulanic acid, as well as inhibition zone diameters of ≤22 mm for cefotaxime and ≤17 mm for ceftazidime, according to CLSI-established breakpoints [15, 16].

*E. coli* American Type Culture Collection (ATCC) 25922 was used as the susceptible quality-control strain for each batch of analyses. In addition, an in-house clinical ESBL-producing *E. coli* isolate, previously confirmed by polymerase chain reaction (PCR) as carrying the *bla*_CTX__-M_*, bla*_SHV_, and *bla*_TEM_ genes, was used as the positive control for both the double-disk synergy test (DDST) and PCR assays.

### Antimicrobial susceptibility testing (AST)

Disk diffusion AST was performed by the Kirby–Bauer method on Mueller–Hinton agar, following CLSI M100 (31st ed., 2021) and CLSI VET01S (5th ed., 2022, veterinary-specific) breakpoints [15, 16]. A panel of 12 antibiotics representing six CLSI-defined antimicrobial categories was tested on each phenotypically confirmed ESBL isolate: β-lactams (cefepime 30 µg, imipenem 10 µg, meropenem 10 µg), aminoglycosides (gentamicin 10 µg, amikacin 30 µg), tetracyclines (tetracycline 30 µg, doxycycline 30 µg, minocycline 30 µg), fluoroquinolones (levofloxacin 5 µg, norfloxacin 10 µg), nitrofurans (nitrofurantoin 300 µg), and folate pathway inhibitors (trimethoprim–sulfamethoxazole 1.25/23.75 µg). Inhibition zone diameters were measured with a digital caliper after 18 h of incubation at 35 ± 2 °C and interpreted as susceptible (S), intermediate (I), or resistant (R) per CLSI M100 31st ed. *E. coli* ATCC 25922 was run in each batch as the quality-control strain. MDR was defined according to Magiorakos *et al.* [17] as acquired non-susceptibility (R or I) to ≥1 agent in ≥3 of the antimicrobial categories listed above.

### Molecular detection of *bla*_CTX__-__M,_*bla*_SHV_, and *bla*_TEM_

Genomic DNA was extracted from fresh overnight subcultures of each confirmed ESBL-producing isolate using the Wizard® SV Genomic DNA Purification System (Promega Corporation, Madison, WI, USA; Cat. No. A2360), according to the manufacturer’s instructions. DNA integrity and concentration were assessed by NanoDrop spectrophotometry, considering an A260/A280 ratio of 1.8–2.0.

Detection of the three target β-lactamase genes was performed using independent PCR reactions. For *bla*_SHV_ and \(bl{a}_{CTX-M}\), the primers described by Dallenne *et al.* [18] were used. The *bla*_SHV_ gene was amplified using primers F: 5′-AGCCGCTTGAGCAAATTAAAC-3′ and R: 5′-ATCCCGCAGATAAATCACCAC-3′, with an expected amplicon of approximately 713 bp. For *bla*_CTX__-M, _primers F: 5′-AATCACTGCGTCAGTTCAC-3′ and R: 5′-TTTATCCCCC ACAACCCAG-3′ were used, generating an expected amplicon of approximately 701 bp. Finally, detection of *bla*_TEM_ was performed using the primer pair described by Maynard *et al.* [19]: F: 5′-GAGTATTCAACATTTTCGT-3′ and R: 5′-ACCAATGCTTAATCAGTGA-3′, with an expected amplicon of approximately 857 bp.

Each PCR reaction was prepared in a final volume of 50 µL, consisting of 34 µL of nuclease-free water, 10 µL of 5× Green GoTaq® Flexi Reaction Buffer, 2 µL of 25 mM MgCl₂, 1.2 µL of dNTPs, 1 µL of each primer, 0.25 µL of GoTaq® DNA polymerase, and 0.55 µL of template DNA.

Amplification was performed in a DLAB TC1000-G-PRO thermal cycler (DLAB Scientific, Beijing, China) under gene-specific conditions. For *bla*_SHV_, the program consisted of initial denaturation at 94 °C for 5 min, followed by 30 cycles of denaturation at 94 °C for 30 s, annealing at 52.8 °C for 30 s, and extension at 72 °C for 1 min, with a final extension at 72 °C for 5 min. For *bla*_CTX__-M, _initial denaturation was performed at 94 °C for 5 min, followed by 30 cycles at 94 °C for 30 s, 54.5 °C for 30 s, and 72 °C for 1 min 50 s, ending with a final extension at 72 °C for 5 min. For *bla*_TEM_, the conditions were as follows: initial denaturation at 94 °C for 5 min, followed by 30 cycles at 94 °C for 30 s, annealing at 51.3 °C for 30 s, and extension at 72 °C for 1 min 50 s, with a final extension at 72 °C for 5 min.

Each PCR run included a no-template negative control consisting only of molecular-grade water and an in-house clinical ESBL-producing *E. coli* isolate, previously confirmed by PCR to carry *bla*_CTX__-M, _*bla*_SHV__,_ and *bla*_TEM_, as the positive control. Amplicons were separated by electrophoresis on 2% agarose gels in Tris-acetate-EDTA buffer stained with GelRed® (Biotium, Fremont, CA, USA), together with a 100 bp Opti-DNA Marker (Applied Biological Materials Inc., abm®, Richmond, BC, Canada; Cat. No. G016) and visualized under UV transillumination using an EDVOTEK model 557 UV transilluminator.

### Risk factor questionnaire

For each cat with a phenotypically confirmed ESBL-producing *E. coli* isolate (n = 83), the owner completed a structured questionnaire administered by a veterinarian. Variables recorded were age (converted to years), sex, breed, owner-reported self-medication with antimicrobials in the prior 6 months, dietary inclusion of raw animal tissue (any frequency), observed coprophagia, previous hospitalization of the cat, and contact with other animals (other cats or dogs, sharing living space or outdoor access).

### Statistical analysis

The association between the presence of cats carrying at least one of the ESBL genes evaluated (*bla*_SHV_, *bla*_CTX-M,_ or *bla*_TEM_) and potential predisposing factors was analyzed using Pearson’s chi-square test or Fisher’s exact test, as appropriate, based on previously constructed contingency tables. The variables considered included age <1 year, self-medication, prolonged antibiotic use, hospitalization, contact with other animals, and raw feeding.

Statistical analysis was performed using SPSS version 25 for Windows. A 95% confidence level and a statistical significance value of α = 0.05 were considered.

## RESULTS

### Study population and prevalence of ESBL-producing *E. coli*

**Figure 1:**
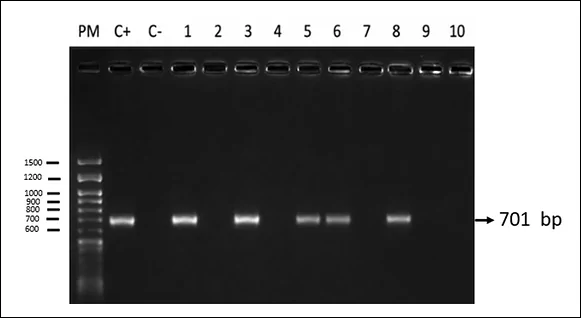
Agarose gel electrophoresis of polymerase chain reaction products for detection of the *bla*_CTX-M_ gene (701 bp) in *Escherichia coli* isolates recovered from domestic cats. PM: Molecular weight marker; C+: Positive control; C−: Negative control; lanes 2, 4, 7, 9, and 10: Negative samples; lanes 1, 3, 5, 6, and 8: Positive samples.

**Figure 2:**
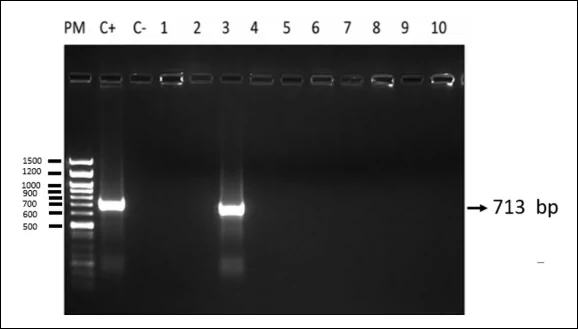
Agarose gel electrophoresis of polymerase chain reaction products for detection of the *bla*_SHV_ gene (713 bp) in *Escherichia coli* isolates recovered from domestic cats. PM: Molecular weight marker; C+: Positive control; C−: Negative control; lanes 1, 2, 4, 5, 6, 7, 8, 9, and 10: Negative samples; lane 3: Positive sample.

**Figure 3:**
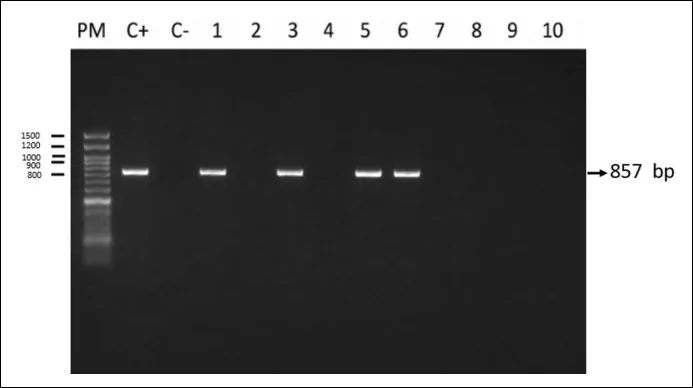
Agarose gel electrophoresis of polymerase chain reaction products for detection of the *bla*_TEM_gene (857 bp) in *Escherichia coli* isolates recovered from domestic cats. PM: Molecular weight marker; C+: Positive control; C−: Negative control; lanes 2, 4, 5, 8, 9, and 10: Negative samples; lanes 1, 3, 6, and 7: Positive samples.

Among the 199 rectal swab samples processed, 83 isolates (41.7%) grew on selective CHROMagar™ ESBL medium and were phenotypically confirmed as ESBL producers using the DDST. Molecular analysis showed a marked predominance of the *bla*_CTX-M_ gene (Figure 1), detected in 60 of 83 isolates (72.3%). In comparison, the *bla*_TEM_ gene (Figure 3) was identified in 19 isolates (22.9%), whereas the *bla*_SHV_ gene (Figure 2) was detected in only one isolate (1.2%).

### Molecular characterization of ESBL genes

PCR targeting the three β-lactamase genes detected at least one target gene in 64 of the 83 isolates (77.1%; 95% Wilson confidence interval [CI]: 66.9%–84.9%), which were subsequently designated as ESBL gene-positive isolates. The remaining 19 isolates (22.9%; 95% CI: 15.1%–33.1%) were phenotypically positive for ESBL production by DDST but were negative for all three target genes by PCR and were therefore classified as ESBL gene-negative isolates.

### Antimicrobial susceptibility profile

**Figure 4:**
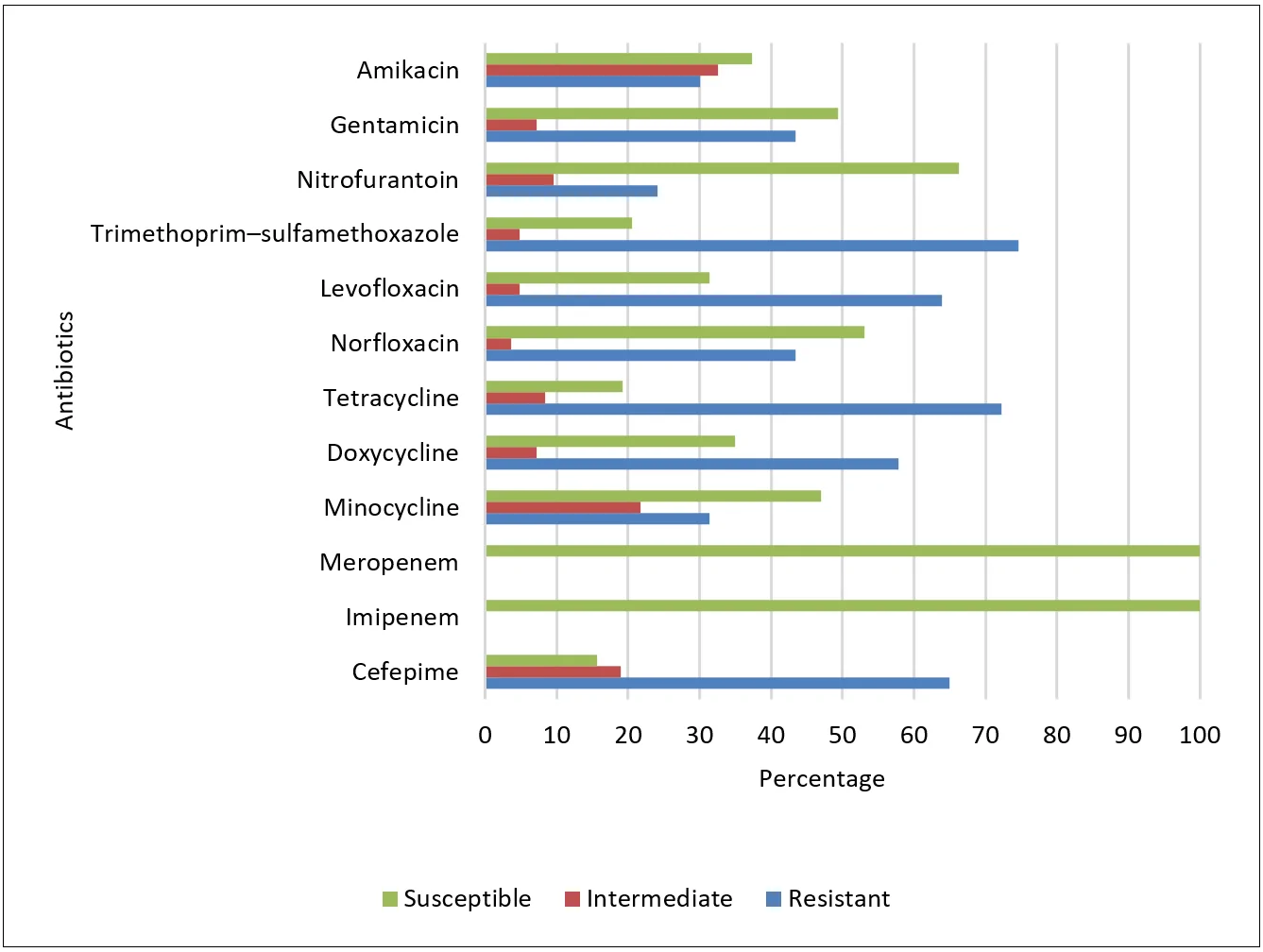
Antimicrobial susceptibility profile of 83 ESBL-producing *E. coli* isolates recovered from domestic cats in Machala, Ecuador, collected between August 2023 and August 2024.

All 83 ESBL-producing *E. coli* isolates were susceptible to the carbapenems meropenem and imipenem (83/83; 100%). The highest resistance rates were observed against trimethoprim–sulfamethoxazole (74.69%), tetracycline (72.28%), cefepime (65.06%), levofloxacin (63.85%), and doxycycline (57.83%). Among the non-carbapenem antimicrobial agents, nitrofurantoin exhibited the highest susceptibility rate (66.26%) (Figure 4).

MDR, defined according to Magiorakos *et al.* [17] as acquired non-susceptibility to at least one antimicrobial agent in three or more antimicrobial categories, was identified in 74 of the 83 isolates (89.2%).

### Risk factor analysis

None of the evaluated risk factors was significantly associated with the presence of the *bla*_CTX-M_, *bla*_SHV_, or *bla*_TEM_ genes (p > 0.05) (Table 1). A higher frequency of ESBL gene-positive isolates was observed among cats older than 1 year of age, those without a history of self-medication or prolonged antimicrobial use, and those with contact with other animals. However, none of these differences were statistically significant based on Pearson's chi-square test or Fisher's exact test.

**Table 1:** Association between owner-reported risk factors and extended-spectrum β-lactamase (ESBL) gene-positive status (carriage of at least one of blaCTX-M, blaSHV, or blaTEM detected by polymerase chain reaction) among 83 phenotypically confirmed ESBL-producing Escherichia coli isolates recovered from domestic cats in Machala, Ecuador, between August 2023 and August 2024.

**Risk factor**	**ESBL gene-positive n = 64 (%)**	**ESBL gene-negative n = 19 (%)**	**p-value**
Age >1 year	49 (76.6)	12 (63.2)	0.245
Age <1 year	15 (23.4)	7 (36.8)	
Self-medication (Yes)	10 (15.6)	6 (31.6)	0.122
Self-medication (No)	54 (84.4)	13 (68.4)	
Prolonged antibiotic use (Yes)	6 (9.4)	5 (26.3)	0.560
Prolonged antibiotic use (No)	58 (90.6)	14 (73.7)	
Hospitalization (Yes)	0 (0.0)	1 (5.3)	0.650
Hospitalization (No)	64 (100.0)	18 (94.7)	
Contact with other animals (Yes)	55 (85.9)	16 (84.2)	0.851
Contact with other animals (No)	9 (14.1)	3 (15.8)	
Raw feeding (Yes)	16 (25.0)	8 (42.1)	0.149
Raw feeding (No)	48 (75.0)	11 (57.9)	
Health status (Healthy)	55 (85.9)	17 (89.5)	0.690
Health status (Sick)	9 (14.1)	2 (10.5)	

### Phenotypically ESBL-positive but PCR-negative isolates

Nineteen isolates (22.9% of the phenotypically confirmed ESBL-producing isolates) were positive by DDST but negative for *bla*_CTX-M_, *bla*_TEM_, and *bla*_SHV_. These findings suggest the presence of β-lactamase genes that were not targeted by the PCR panel described by Dallenne *et al.* [18], including CIT/FOX-family AmpC β-lactamases, OXA-1-like enzymes, minor *bla*_CTX-M_ groups (e.g., CTX-M-2), and GES/PER-family β-lactamases. Comprehensive characterization of these isolates using whole-genome sequencing or broader PCR panels, such as the AmpC PCR panel described by Pérez-Pérez and Hanson [20] or the ESBL detection panel developed by Ellington *et al.* [21], represents a limitation of the present study. All 19 DDST-positive/PCR-negative isolates have been preserved as 2% glycerol cryostocks at −80°C and are scheduled for whole-genome sequencing during the second phase of the project under the same institutional research framework at the Universidad Técnica de Machala.

## DISCUSSION

### ESBL prevalence in the context of Latin America and companion animals

The present study demonstrated an intestinal carriage prevalence of ESBL-producing *E. coli* of 41.7% (83/199) among domestic cats in Machala, Ecuador. This prevalence is among the highest reported for feline populations worldwide and exceeds those documented in companion animal studies from neighboring Andean countries, including Ecuador [12] and Peru [22], as well as reports from Europe and Asia [9, 23, 24]. Nevertheless, the observed prevalence should be interpreted within the context of the study design. Sampling was conducted at a referral Veterinary Clinic, where the patient population is likely to differ from that of the general community in age, health status, and prior antimicrobial exposure. Referral clinics commonly receive animals with underlying illnesses or recent antimicrobial treatment, both of which are important factors associated with intestinal colonization by ESBL-producing *E. coli* [7].

Despite this potential selection bias, the remarkably high prevalence observed in the present study is unlikely to be explained solely by referral bias or clinical amplification. Instead, it strongly suggests widespread community circulation of ESBL-producing *E. coli* among owned cats in the study area. This interpretation is biologically plausible because El Oro Province is characterized by intensive pig, poultry, and shrimp production systems, which have previously been associated with environmental dissemination of ESBL-producing bacteria through contaminated food products, water sources, and occupational exposure in coastal Ecuador [25]. Companion cats living within this ecosystem may acquire resistant bacteria through multiple environmental pathways, including contaminated water, locally produced commercial pet food, hunting rodents and birds, or indirect contact with contaminated environments, thereby facilitating the continuous circulation of resistant strains among humans, animals, and the environment within a One Health context.

### Distribution of ESBL genes and predominance of *bla*_CTX-M_

Among the 83 phenotypically confirmed ESBL-producing isolates, *bla*_CTX-M_ was the predominant gene, being detected in 60 isolates (72.3%), whereas *bla*_TEM_ and *bla*_SHV_ were identified in 19 (22.9%) and 1 (1.2%) isolates, respectively. The predominance of *bla*_CTX-M_ is consistent with the current global epidemiology of ESBL-producing *E. coli* described by Bevan *et al.* [2] and corroborates findings from companion animal surveillance studies conducted in Latin America, particularly Brazil [10], as well as pooled international meta-analyses involving dogs and cats [7, 11].

The very low prevalence of *bla*_SHV_ observed in this study further supports the progressive global replacement of SHV-type ESBLs by CTX-M enzymes during the last two decades [4, 26]. This epidemiological shift has been attributed to the exceptional mobility of *bla*_CTX-M_ genes, which are frequently associated with highly transferable plasmids and insertion sequences that facilitate rapid horizontal dissemination among *Enterobacterales*. Consequently, *bla*_CTX-M_ has become the dominant ESBL determinant in both human and veterinary medicine worldwide.

Although less frequently detected, the presence of *bla*_TEM_ in nearly one-quarter of the isolates suggests continued circulation of TEM-family β-lactamases within companion animal populations in Ecuador. These findings are consistent with previous reports describing co-circulation of TEM-type enzymes, including TEM-1 and ESBL-associated variants such as TEM-52, in companion animals from Latin America [12]. The coexistence of *bla*_CTX-M_ and *bla*_TEM_ within the same ecological niche further highlights the complexity of ESBL epidemiology and emphasizes the role of companion animals as reservoirs of multiple resistance determinants that may be exchanged among bacterial populations through horizontal gene transfer.

### Antimicrobial susceptibility and MDR

The antimicrobial susceptibility profile revealed an alarmingly high prevalence of MDR, with 74 of the 83 ESBL-producing isolates (89.2%; 95% CI: 80.7%–94.2%) classified as MDR according to the criteria proposed by Magiorakos *et al.* [17]. This MDR frequency exceeds that reported in previous companion animal studies from Ecuador [12, 27] and Brazil [10] and approaches the resistance levels documented among hospital-associated ESBL-producing *E. coli* isolated from human clinical infections in coastal and Andean Ecuador [25]. These findings suggest that companion animals may increasingly participate in the same AMR ecosystem as humans, reinforcing the importance of integrated One Health surveillance.

Of particular clinical importance, all isolates remained susceptible to the carbapenems imipenem and meropenem, confirming that these agents remain effective last-line therapeutic options for severe infections caused by ESBL-producing *E. coli* in companion animals [28]. Preservation of carbapenem susceptibility is encouraging; however, it also emphasizes the importance of prudent antimicrobial stewardship to prevent the emergence and dissemination of carbapenem-resistant organisms within veterinary settings.

Among the non-carbapenem agents evaluated, nitrofurantoin and minocycline retained comparatively greater antimicrobial activity, with resistance rates of 24.1% and 31.3%, respectively. Likewise, amikacin demonstrated relatively favorable activity despite resistance being observed in 30.1% of isolates and intermediate susceptibility in an additional 7.2%. These findings suggest that these antimicrobials may remain useful therapeutic alternatives for selected uncomplicated infections when treatment is guided by AST and interpreted in accordance with current CLSI recommendations. Nevertheless, empirical use should be approached cautiously because of the substantial MDR burden observed in the present study.

The exceptionally high frequency of MDR among ESBL-producing isolates further suggests that resistance is not solely attributable to the presence of individual ESBL genes but rather reflects dissemination of complex mobile genetic elements carrying multiple resistance determinants. In particular, plasmids belonging to the IncF and IncI1 incompatibility groups commonly harbor *bla*_CTX-M_ together with additional resistance genes, including *qnr*, *aac*(6′)-*Ib-cr*, *tet(A)*, and *sul*, thereby promoting co-selection and long-term persistence of multidrug-resistant phenotypes under antimicrobial selection pressure [29, 30]. The widespread dissemination of these MDR plasmids likely contributes substantially to the resistance profiles observed in the present study and further supports the hypothesis of extensive community-level circulation of resistant *E. coli* strains rather than isolated emergence within individual animals.

### Absence of risk factor associations and epidemiological saturation

None of the evaluated owner-reported risk factors showed a statistically significant association with ESBL gene-positive status. However, the absence of statistically significant associations should not be interpreted as evidence that these factors are epidemiologically unimportant. Rather, the findings may reflect a state of epidemiological saturation, in which a resistance determinant has become sufficiently widespread within a population that conventional risk factors lose their discriminatory capacity. Under such circumstances, both apparently exposed and unexposed animals may already be colonized, thereby reducing the ability of cross-sectional analyses to detect significant differences between groups.

This interpretation is further supported by the analysis according to clinical health status. The proportion of apparently healthy cats was comparable between the ESBL gene-positive subgroup (55/64; 85.9%) and the phenotypically ESBL-positive but PCR-negative subgroup (17/19; 89.5%), with no statistically significant difference (p = 0.690; Table 1). The predominance of apparently healthy animals in both groups indicates that intestinal carriage of ESBL-producing *E. coli*, predominantly associated with *bla*_CTX-M_, is not confined to clinically ill cats. Consequently, carriage is unlikely to be driven solely by recent disease or acute antimicrobial exposure. Instead, the findings are more consistent with widespread community-level transmission, in which neither health status nor individual owner-reported clinical exposures independently predict ESBL carriage.

In settings with lower ESBL prevalence, conventional risk factors such as previous hospitalization, recent antimicrobial use, and contact with other animals have frequently been associated with colonization by ESBL-producing *E. coli* [8, 31. In contrast, the present study identified an overall ESBL prevalence of 41.7%, while 89.2% of ESBL-producing isolates exhibited MDR. These findings indicate that resistant bacteria are already widely disseminated within the study population, thereby limiting the explanatory value of traditional epidemiological risk factors. Collectively, these observations suggest that community-wide transmission, rather than isolated exposure events, is the principal driver of ESBL dissemination among domestic cats in Machala.

### Strengths and limitations

The present study has several strengths. To our knowledge, it represents the first molecular epidemiological investigation of intestinal ESBL-producing *E. coli* in domestic cats from Ecuador. Furthermore, the combined use of phenotypic confirmation, molecular characterization of the principal ESBL genes, comprehensive AST, and evaluation of epidemiological risk factors provides a broad overview of ESBL dissemination in companion cats within a One Health context.

Nevertheless, several limitations should be acknowledged. First, the study was conducted at a single referral Veterinary Clinic, which may limit the generalizability of the findings to the wider feline population of El Oro Province and Ecuador. Second, although ESBL production was phenotypically confirmed using the DDST according to CLSI recommendations, determination of the minimum inhibitory concentration (MIC) or E-test confirmation was not performed. Inclusion of these quantitative methods would have provided a more detailed assessment of antimicrobial susceptibility.

Third, the PCR assays identified the major ESBL gene families but did not differentiate individual variants, such as CTX-M-15 from CTX-M-14 or TEM-1 from TEM-52. Consequently, the genetic diversity and evolutionary relationships of the circulating ESBL determinants could not be fully characterized. Identification of these variants, together with investigation of clonal relationships, would require Sanger sequencing or whole-genome sequencing. Finally, the molecular screening panel did not include AmpC, OXA-1-like, GES, or PER β-lactamases, which may explain the 19 phenotypically positive but PCR-negative isolates. Future investigations should therefore incorporate broader molecular approaches, including whole-genome sequencing, multilocus sequence typing (MLST), plasmid replicon typing, phylogenetic analysis, and comprehensive β-lactamase gene screening to better elucidate the epidemiology and transmission dynamics of ESBL-producing *E. coli* in companion animals.

### Implications for One Health and potential transmission pathways

The combination of a high prevalence of ESBL-producing *E. coli* (>40%) and an exceptionally high prevalence of MDR (approximately 90%) among colonized cats has important implications that extend beyond companion animal medicine. In a region where formal antimicrobial stewardship programs in small-animal practice remain limited, these findings support the existence of an established community reservoir of antimicrobial-resistant bacteria. Several plausible and potentially overlapping transmission pathways may contribute to this epidemiological pattern.

One potential pathway is household microbiome sharing. Domestic cats frequently live in close physical contact with their owners, share sleeping areas, receive food directly from human hands, and engage in grooming behaviors that facilitate dissemination of fecal bacteria onto fur and household surfaces. These interactions provide repeated opportunities for bidirectional exchange of *E. coli* strains between the intestinal microbiota of humans and companion animals [8, 32]. Environmental contamination within households may further facilitate bacterial persistence and transmission. For example, ESBL-producing *E. coli* has been isolated from both cats and their drinking water in Thai households, where *bla*_TEM_ was detected in 88.7% of isolates and closely related AMR profiles were observed across the cat–environment interface [33]. Similarly, carriage of *bla*_CTX-M_-positive *E. coli* in companion animals and associated resistance plasmids has been documented in regional studies, supporting the possibility of bidirectional transmission of pandemic *bla*_CTX-M_ lineages between humans and companion animals, as previously reported in Quito, Ecuador [12].

A second potential transmission pathway involves environmental and food-chain reservoirs. El Oro Province is characterized by intensive pig, poultry, and shrimp production, and previous studies have demonstrated the presence of ESBL-producing *Enterobacterales* in ready-to-eat foods, irrigation water, and aquaculture effluents within coastal Ecuador [25, 34, 35]. Cats with outdoor access may therefore acquire ESBL-producing *E. coli* through ingestion of contaminated water, predation on wildlife, or consumption of contaminated food sources. Furthermore, self-transmissible plasmids belonging to the IncF and IncI1 incompatibility groups commonly harbor *bla*_CTX-M_ together with additional resistance genes, including *qnr*, *aac*(6′)-*Ib-cr*, *tet(A)*, and *sul*, thereby facilitating co-selection and maintenance of multidrug-resistant strains within both animal and environmental reservoirs [29, 30].

Taken together, the convergence of high ESBL prevalence, extensive MDR, and the absence of significant associations with conventional risk factors strongly supports a pattern of community-driven AMR endemicity rather than transmission confined to clinical settings [36]. Under this scenario, domestic cats should not be regarded merely as passive sentinels of AMR but as active participants within a complex local One Health network linking humans, companion animals, livestock, wildlife, food systems, and the environment.

From a practical perspective, these findings support several priority actions. First, companion animals should be incorporated into Ecuador's national AMR surveillance framework to complement existing human and livestock surveillance activities [2]. Second, evidence-based antimicrobial stewardship guidelines for small animal practice should be developed using regional antimicrobial susceptibility data. Such measures are particularly important because inappropriate owner-initiated antimicrobial administration remains common throughout Latin America, where over-the-counter access to broad-spectrum antimicrobials has been documented in veterinary practice, including the first national veterinary prescribing survey conducted in Chile [37–39]. Although quantitative data on owner-driven antimicrobial self-medication in Ecuador remain limited, the present study observed frequent owner-initiated antimicrobial use prior to veterinary consultation. This practice represents an important and potentially modifiable target for future antimicrobial stewardship interventions.

## CONCLUSION

This study provides the first molecular epidemiological evidence of intestinal ESBL-producing *E. coli* carriage in domestic cats in Ecuador and demonstrates a high prevalence of ESBL-producing isolates (41.7%) in the study population. Molecular characterization revealed a clear predominance of *bla*_CTX__-M_, followed by *bla*_TEM_ and *bla*_SHV_, while nearly one-quarter of the phenotypically confirmed ESBL-producing isolates lacked the targeted ESBL genes, indicating the probable circulation of additional β-lactamase families. Furthermore, MDR was detected in 89.2% of isolates, while complete susceptibility to imipenem and meropenem was maintained, underscoring the continued effectiveness of carbapenems against these isolates.

The absence of statistically significant associations between ESBL carriage and the evaluated owner-reported risk factors, together with the high prevalence of resistant isolates among apparently healthy cats, suggests that ESBL-producing *E. coli* is widely disseminated within the community rather than being confined to animals with specific clinical or management-related exposures. These findings reinforce the importance of companion cats as potential reservoirs of antimicrobial-resistant bacteria within the human–animal–environment interface and highlight their relevance in One Health surveillance programs.

A major strength of this study is that it represents the first comprehensive molecular investigation of ESBL-producing *E. coli* in domestic cats from Ecuador, combining phenotypic confirmation, molecular detection of the major ESBL genes, AST, and epidemiological risk factor analysis. However, the findings should be interpreted with several limitations in mind, including sampling from a single referral Veterinary Clinic, the absence of MIC determination and sequencing-based characterization, and the lack of molecular investigation of additional β-lactamase families, plasmid types, and bacterial lineages.

Future studies should expand surveillance across multiple geographic regions and include whole-genome sequencing, MLST, plasmid replicon analysis, and integrated sampling of humans, companion animals, livestock, food products, and environmental sources to better define transmission pathways and the dynamics of AMR dissemination. Longitudinal investigations evaluating antimicrobial use, environmental exposure, and household transmission would further improve understanding of the epidemiology of ESBL-producing *E. coli* in companion animals.

Overall, the high prevalence of *bla*_CTX-M_-dominant ESBL-producing *E. coli* and the widespread MDR identified in domestic cats underscore the need to incorporate companion animals into Ecuador's national AMR surveillance and antimicrobial stewardship programs. Such integrated One Health approaches will be essential for limiting the dissemination of antimicrobial-resistant bacteria and preserving the effectiveness of critically important antimicrobials for both veterinary and human medicine.

## DATA AVAILABILITY

The supplementary data can be made available from the corresponding author upon request.

## AUTHORS’ CONTRIBUTIONS

MG: Conceived and designed the study, performed the laboratory investigation and molecular analyses, analyzed and interpreted the data, drafted the manuscript, and critically revised the manuscript. SR, AR, VR, GK, PN, LJ, GJ, and RS: Contributed to the study design, laboratory investigation, data interpretation, and critical revision of the manuscript. All authors have read and approved the final manuscript.
